# The commercial performance of cellulosic ethanol supply-chains in Europe

**DOI:** 10.1186/1754-6834-2-3

**Published:** 2009-02-03

**Authors:** Raphael Slade, Ausilio Bauen, Nilay Shah

**Affiliations:** 1 Imperial Centre for Energy Policy and Technology, Centre for Environmental Policy, Imperial College London, South Kensington Campus, London SW7 2AZ, UK; 2 Centre for Process Systems Engineering, Imperial College London, South Kensington Campus, London SW7 2AZ, UK

## Abstract

**Background:**

The production of fuel-grade ethanol from lignocellulosic biomass resources has the potential to increase biofuel production capacity whilst minimising the negative environmental impacts. These benefits will only be realised if lignocellulosic ethanol production can compete on price with conventional fossil fuels and if it can be produced commercially at scale. This paper focuses on lignocellulosic ethanol production in Europe. The hypothesis is that the eventual cost of production will be determined not only by the performance of the conversion process but by the performance of the entire supply-chain from feedstock production to consumption. To test this, a model for supply-chain cost comparison is developed, the components of representative ethanol supply-chains are described, the factors that are most important in determining the cost and profitability of ethanol production are identified, and a detailed sensitivity analysis is conducted.

**Results:**

The most important cost determinants are the cost of feedstocks, primarily determined by location and existing markets, and the value obtained for ethanol, primarily determined by the oil price and policy incentives. Both of these factors are highly uncertain. The best performing chains (ethanol produced from softwood and sold as a low percentage blend with gasoline) could ultimately be cost competitive with gasoline without requiring subsidy, but production from straw would generally be less competitive.

**Conclusion:**

Supply-chain design will play a critical role in determining commercial viability. The importance of feedstock supply highlights the need for location-specific assessments of feedstock availability and price. Similarly, the role of subsidies and policy incentives in creating and sustaining the ethanol market highlights the importance of political engagement and the need to include political risks in investment appraisal. For the supply-chains described here, and with the cost and market parameters selected, selling ethanol as a low percentage blend with gasoline will maximise ethanol revenues and minimise the need for subsidies. It follows, therefore, that the market for low percentage blends should be saturated before markets for high percentage blends.

## Background

Globally, ethanol and biodiesel are the most widely available biofuels, and in response to politically stimulated demand, production capacity has grown rapidly. As production increases, however, concerns about the negative environmental and social impacts of biofuels are also on the rise. In particular, there are fears that competition between fuel and food crops will lead to higher food prices; that production of biofuel feedstocks will accelerate the expansion of agriculture thereby initiating direct and indirect land use change (for example, loss of grasslands and forests); and that carbon dioxide emissions associated with land use change may negate any benefits obtained from fossil fuel substitution [1,2].

The production of biofuels from lignocellulosic biomass resources has the potential to increase biofuel production capacity whilst minimising the negative environmental and social impacts because lignocellulosic resources (for example, forestry residues, wheat straw, corn stover, etc.) do not compete directly with food production, or with land that may be needed for food production. Using these resources efficiently, however, requires conversion technologies such as advanced hydrolysis and fermentation to produce ethanol – the focus of this paper – or the production of synfuels via gasification. These technologies are currently pre-commercial and face technical, economic and political hurdles. Public finance and political support can help tackle these hurdles, thereby accelerating technical development and deployment, but, in the words of one UK policy-maker: "biofuels do not have an automatic right to support, just because people are enthusiastic" D Vincent, Carbon Trust, personal communication, 2006. The case for support must be justified on the basis of the future technical and market potential.

This paper focuses on the production of ethanol from lignocellulosic feedstocks in Europe, and investigates the factors that will determine its eventual cost of production and commercial viability. The hypothesis is that if lignocellulosic ethanol (LE) is to be produced commercially at scale then the eventual cost of production will be determined not only by the performance of the conversion process but by the performance of the entire supply-chain from feedstock production to consumption. To investigate this, a holistic cost model has been developed permitting the rapid comparison of different process concepts at the supply-chain level. This model uses simplified descriptions of LE conversion processes, together with feedstock and ethanol price estimates and finance scenarios, to determine the sensitivity of the production cost to changes in the supply-chain.

The paper is presented in three parts. The first part reviews previous cost estimates, identifies the supply-chains of greatest interest in Europe, defines a basis for supply-chain cost comparison, and describes the basic structure of the model. The second part describes the components of representative ethanol supply-chains. It identifies generic values for the most important parameters affecting cost performance, as well as the range of values that they may take. Lastly, base-case supply-chains are proposed and compared and a detailed sensitivity analysis is presented. The aspects of the supply chain that have the greatest influence on the cost of ethanol production are identified, and the implications, in terms of the future commercial viability of lignocellulosic ethanol, are discussed.

### Identifying representative supply-chains and defining a basis for comparison

#### Cost assessments in the academic literature

Numerous cost estimates can be found in the literature, but the majority of these focus exclusively on the conversion process and use production cost as a simple metric to compare alternative process designs. A typical example is the process design and economic analysis approach used by the US National Renewable Energy Laboratory (NREL) [3]. This approach estimates process yields, material flows and capital equipment requirements, using experimental data and a flow-sheeting software package (Aspen Plus™). The cost of capital equipment is estimated using vendor quotations or data from a cost database (for example, ICARUS™). The minimum cost of ethanol is then determined using a discounted cash flow analysis.

This is an accepted approach to process development, but the emphasis on the conversion process ignores, or sets as constant, many of the other factors that may influence overall commercial viability and supply-chain performance. For example, the way in which a plant is financed, its size and location, its potential for technological progress, and the issues associated with securing a reliable source of biomass, may all affect its commercial viability, and may change as the technology develops. It is our view that these factors are likely to be more important for biofuel processes than for typical petroleum refinery or petrochemical processes, which work with standardised equipment and commodity, high energy density feedstocks.

Cross comparison between studies is also problematic. To illustrate this point, recently published cost estimates are summarised in Table 1[4-8], normalised to 2005 US$. It can be seen that studies conducted in the US (NREL, Lynd) generally forecast a lower cost of ethanol than those conducted in the EU (Von Sivers, Wingren, Sassner). In part, this is due to the US studies assuming a conversion plant more than twice as large as that assumed in the EU studies, but there are numerous other assumptions embedded in the estimates which are less transparent, for example, feedstock cost assumptions, process yields, co-product values, etc.

**Table 1:** Cost estimates for lignocellulosic ethanol production

**Reference**	**Conversion process^a^**	**Capacity** (tonnes dry biomass year^-1^)	**Ethanol production cost** (_2005_US$ litre^-1^)
Von Sivers and Zacchi (1996) [4]	Enz./dilute acid/concentrated acid	100,000 (softwood)	0.76/0.81/0.79

Lynd (1996) [5]	Enz. (SSF)	592,000 (hardwood)	0.4

National Renewable Energy Laboratory: Wooley et al. (1999) [3] Aden et al. (2002) [6] Ruth and Jechura (2003) [7]	Enz. (SSF)	700,000 (hardwood)/(corn stover)	0.47/0.34

Wingren (2003) [8]	Enz. (SHF)/Enz. (SSF)	196,000 (softwood)	0.8/0.68–0.64

Sassner et al. (2008) [26]	Enz. (SSF)	200,000 (hardwood/corn stover/softwood)	0.71/0.71/0.57

^a^Process classified according to principal hydrolysis step: Enz. = enzymatic hydrolysis; SSF = simultaneous saccharification and fermentation; SHF = separate hydrolysis and fermentation.

Estimates are normalised for currency, year and units.

#### Selecting European supply-chains for evaluation

At the highest level, the components of the lignocellulose-to-ethanol supply-chain are generic: feedstock supply, conversion, and distribution and utilisation. At lower levels of aggregation the options diversify rapidly and many alternative supply-chains can be conceptualised. This paper, however, focuses on a limited number of supply-chains. Specifically, ethanol produced from softwood or wheat straw, using a dilute acid or enzymatic conversion process, and distributed as either a 5% (E5) or 85% (E85) blend with gasoline. These supply-chains are amongst those with the greatest potential within Europe. Softwood and forest fuels are of interest because of their abundance (around 411 TWh year^-1 ^[9]) in northern Europe and the low input intensity of silviculture compared with agriculture. Straw is of interest because of its abundance as a co-product of cereal production (around 63–227 TWh year^-1^[10]), and relatively low cost.

Reflecting the interest in softwood and straw as feedstocks, conversion processes for these materials are actively being investigated by European companies and universities. They are also the focus of a large EU research project: 'New Improvements in Lignocellulosic Ethanol (NILE)' . Sweden leads the development of softwood conversion: a soft-wood-to-ethanol pilot plant is operated by Sekab (a Swedish company that imports, upgrades, blends and distributes ethanol to serve the Swedish transport market [11]); process development is also undertaken at the University of Lund. Research on the conversion of straw to ethanol is somewhat less advanced, but is the subject of ongoing research at the French Institute for Petroleum and Dong Energy (a Danish energy company) amongst others.

Distribution systems have already been established in Europe using imported ethanol from Brazil and some regionally produced ethanol from cereals, sugar beet and wine surpluses. The majority of ethanol is distributed as E5. E85 is also available in Sweden at around 25% of service stations [11], and on a more limited trial basis in other EU countries.

## Methods

### Developing a supply-chain cost model

#### Defining a basis for comparison

**Figure 1:**
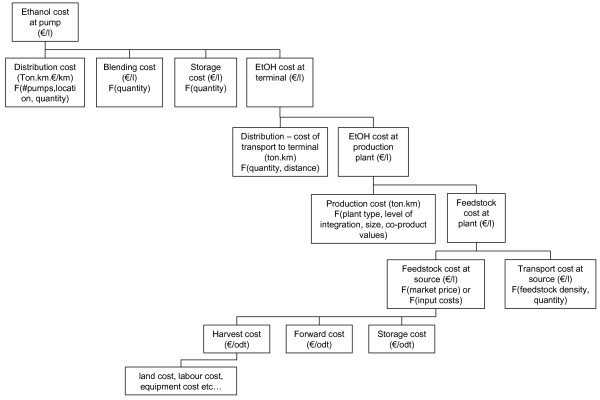
**The ethanol supply-chain cost hierarchy**.

For this analysis, the end-point of the ethanol supply-chain was considered to be the cost of ethanol at the pump, excluding tax. The starting point was the market price of biomass feedstocks and the estimated cost of other inputs (capital, chemicals etc.). Whilst it would theoretically be possible to extend the feedstock part of the supply-chain to include silvicultural (or agricultural) inputs such as the cost of land, cost of harvesting machinery, etc., this approach was rejected as markets already exist for the majority of biomass feedstocks. Feedstock price, as currently determined by the interaction of demand and supply, was therefore judged a better reflection of the situation faced by a prospective project developer than the sum of input costs. The principal cost elements of the bio-ethanol supply-chain are illustrated in Figure 1. This cost hierarchy illustrates that, at each stage of the supply-chain, the cost of intermediates is the sum of the cost of logistics plus the cost of conversion from one product to another.

#### Quantifying supply-chain cost performance

Two measures of supply-chain cost performance were selected: net present value (NPV) and the levelised cost per litre; both are calculated from a discounted cash flow analysis of supply-chain costs and revenues. The NPV is the sum of discounted cash flows, and the more profitable the supply-chain the greater the NPV. NPV provides a comprehensive measure of project value and permits variables such as the tax rate, time taken to build the plant, etc., to be included in the analysis. The disadvantage of this measure is that it does not intuitively relate to other real world quantities such as the oil price. The levelised cost per litre is the ethanol price at which the supply-chain begins to be profitable (has an NPV of zero). The advantage of this measure is that it allows supply-chain performance to be readily related to the cost of alternatives.

Using these cost metrics, a supply-chain can be considered viable if the NPV is greater than zero for a given discount rate. Likewise, ethanol can be considered competitive (without any policy interventions such as renewable obligations, subsidies or carbon taxes) with gasoline if its levelised cost is equal to, or lower than, the wholesale price of gasoline. Caution, however, is required on two counts. Firstly, it does not follow that a project which has an NPV greater or equal to zero is necessarily an attractive investment; different investors apply different discount rates depending upon their assessment of project risk and usually evaluate projects as part of a portfolio rather than in isolation. Secondly, the market value of ethanol is determined by the subsidy regime, its value as an oxygenate, and whether it is sold as E5 or E85. It is therefore possible for the levelised cost per litre of ethanol to be greater than the cost of a litre of gasoline and for the supply-chain to be profitable.

#### Cost model schematic

**Figure 2:**
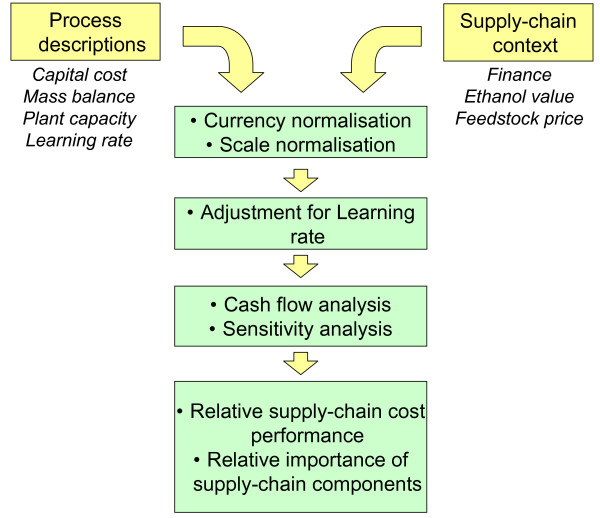
**Supply-chain cost model schematic**.

The supply-chain cost model developed here is a spreadsheet-based tool incorporating a macro-driven sensitivity analysis. The model is shown schematically in Figure 2. The inputs are twofold: firstly, descriptions of the conversion plant and process (mass balance, capital cost, plant capacity and learning rate); and secondly, descriptions of the supply-chain context (feedstock prices, ethanol value, finance package). The outputs, as described above, are estimates of levelised cost and NPV.

The key difference between this model and the flow-sheeting approach used by NREL is that, in the NREL model, the majority of supply-chain parameters are fixed to allow different process designs to be investigated; in our model, the mass balance and equipment structure of each process description are fixed to allow the importance of the other supply-chain parameters to be investigated. There is, in principal however, no limit to the number of supply-chains that could be described and compared, thus a comprehensive picture may be obtained by using a series of mass balance estimates.

#### Model validation

Our model is, in essence, a summation of cost estimates in which numerous assumptions and decisions are embodied. It cannot be tested empirically, but to ensure the overall results are representative, multiple estimates for input parameters were used, taken from a wide range of published and peer-reviewed sources. Assumptions were also discussed with experts from within the NILE project and were moderated accordingly. The sensitivity analysis embodied within the model also helps to identify those areas where greatest precision is necessary.

### Characterising European supply-chains

This section describes the principal components of generic LE supply-chains. Representative values for the most important parameters affecting cost performance are identified and normalised.

#### Characterising feedstock parameters

##### Approach and assumptions

Estimates of biomass costs were identified in reports from national governments, the EU, trade associations and the academic literature. For established biomass resources, such as pulplogs, market indices were also available. One of the consequences of using such a diverse range of sources was that estimates were highly variable in terms of units, currency, extent of processing, location and biomass form such as chips, logs, etc. To enable estimates to be compared on a similar basis, each estimate was normalised using the following assumptions.

• The feedstock supply-chain was assumed to be composed of three generic operations:

○ production and forwarding to a roadside collection point;

○ transport from the roadside collection point to the plant;

○ size reduction, where required, in order that the biomass is in a form acceptable to the plant.

• The conversion plant was assumed to only receive chips or, in the case of the straw process, bales.

• Biomass was transported in the densest form possible, for example, for logs, chipping at the plant was given preference to chipping at the roadside.

• Where estimates did not include transport (and/or size-reduction), a uniform cost was assumed, determined by the biomass form and country of origin as outlined in Table 2[12,13].

**Table 2:** Average transport cost assumptions for feedstock price normalisation

**Operation**	**Country of origin**	**Biomass form**	**Average cost** _2005_US$ odt^-1^	**Comment**	**Reference**
Transport	Sweden	Logs	11.6	Average Scandinavian transport cost assuming 107 km trip	[12]
		
	UK	Logs	21.7	Average UK transport cost assuming 107 km trip	
		
	UK/Sweden	Chips	14.0	Average transport cost assuming 50 km trip	[22]
		
	International	Chips	25.0	1500 km trip – non-dedicated ship	
				
		Bundles	44.3		
			
		Logs	47.7	10,000 km trip – non-dedicated ship	
		
	UK	Bales (straw)	14.9	Average transport cost assuming 50 km trip	[13]

Size reduction	UK Sweden	All	3.7	Hammermill – 12 month operation window	[22]

• Quantities were converted to oven-dry-tonne (odt) equivalents. Prices were converted to 2005 US$.

• Prices were assumed not to vary with the quantity demanded.

Total feedstock costs were then calculated as the sum of cost at roadside, transport cost and size reduction cost.

Given the diversity of potential feedstock supply-chains, it should be recognised that these assumptions represent a simplification. Nevertheless, the level of resolution corresponds with the quality and availability of data and is consistent with the analytical methods underpinning a number of UK government reports including the recent UK biomass strategy [14].

##### Selecting representative feedstock cost estimates

Using the normalising assumptions above, a survey of feedstock prices in Europe was undertaken for straw, waste paper, energy crops, imported biomass, mill residues, forest residues, pulplogs and sawlogs. Cost estimates varied widely for all the types of feedstocks considered. Focusing on the feedstocks expected to be used in the short and medium term: the cost of chips derived from pulplogs varied from US$59 to US$120 odt^-1^, whereas chips derived from forest residues varied from US$38 to US$172 odt^-1^; the cost of mill waste was US$107 to US$108 odt^-1 ^and straw US$66 to US$153 odt^-1^. Looking at the costs of other feedstocks: energy crops varied from US$65 to US$172 odt^-1^; the cost of waste paper showed a considerable range, US$65 to US$172 odt^-1 ^depending upon the quality of the paper: the low estimate shown here corresponds to mixed waste, the higher estimate for newsprint; sorted office paper would fetch a higher price still [15-25].

Given that the variation in prices between resource types was comparable to the variation within each type, we decided not to differentiate between resources, but to use mid-point, high and low cost values as inputs to the model. Mid-point values corresponding to the geometric mean were chosen with high and low estimates corresponding to the 15th and 85th percentiles respectively (that is, approximately one standard deviation from the mean), see Table 3.

**Table 3:** Normalised feedstock cost estimates

**Feedstock **(delivered to the plant as chips/bales)	**Cost **(_2005_US$ odt^-1^)		
	
	**Low**	**Mid**	**High**
Softwood^a^	51.5	74.5	107.7

Straw	66.1	100.9	153.9

^a^Mid-point is equal to geometric mean of pulplog and forest residue costs only. The geometric mean was chosen because plotting a histogram of estimates indicated a log normal distribution.

##### Characterising the conversion process

The economic performance of the conversion process was determined by three groups of variables.

• The capital cost, plant size and level of technological learning attained.

• Product yields, associated flows of materials and energy (chemicals, feedstocks, etc.) and their corresponding unit costs and revenues.

• Cost of finance.

Yields and material flows are interdependent; consequently, detailed investigation of a specific conversion process would typically use a flow-sheeting model to solve the mass and energy balances and identify the impacts of process alterations. Developing new process models for all the conversion processes of interest, however, was beyond the scope of this study, and would entail a level of resolution in excess of the minimum required for the high-level comparison of supply-chain systems. Instead, data from pre-existing process models was used, and where necessary, adapted. For each process configuration the mass balance was fixed and assumed to be invariant with changes in plant capacity. Capital costs were adjusted to reflect plant capacity using a scale relationship, see 'incorporating scale effects' below. The unit cost of inputs, the cost of finance and other parameters could then be varied.

The cost of finance was considered to be an independent variable, principally determined by the market cost of capital and investors' appetite for risk.

##### Describing a reference-case conversion processes for comparison

Process data for enzymatic and dilute acid processes utilising softwood was obtained from the University of Lund [26]. The data was derived from Aspen Plus models of a 25 odthour^-1 ^(around 55 million litres year^-1^ethanol) stand-alone facility, validated by laboratory scale experiment. Costs were determined using ICARUS™ process evaluator, vendor quotations and literature estimates. The enzymatic plant cost 134 million _2005_US$ and employed single-step steam-and-sulphur-dioxide catalysed pre-treatment followed by simultaneous saccharification and fermentation (SSF) using commercially purchased enzymes and yeast produced in the plant; solid fuel (lignin) was exported and sold. The dilute acid plant cost 150 million_2005_US$ and the process was similar to the enzymatic process, except that separate hydrolysis and fermentation (SHF) was undertaken using a two-stage acid-catalysed pre-treatment and hydrolysis step. Neither process included pentose fermentation, this is considered further below. Detailed mass balance data, together with cost assumptions for each process are shown in Table 4.

**Table 4:** Mass balance and costs assumptions for softwood reference conversion processes

**Feedstock/co-product cost**			**Mass balance**									
			**Enzymatic process**					**Dilute acid process**				
	**Units**	**Cost/value **(_2005_US$ unit^-1^)	Input (unit odt^-1^)	Output (unit odt^-1^)				Input (unit odt^-1^)	Output (unit odt^-1^)			
						
				**Ethanol**	**CO_2_**	**Solid fuel**	**Waste** (solid + liquid)		**Ethanol**	**CO_2_**	**Solid fuel**	**Waste** (solid + liquid)

**Biomass**	kg		620									
Hexose				219	245		156	620	173	190		257
Pentose			60				60	60				60
Lignin			280			252	28	280			273	7
Other			40				40	40				40

**Chemicals**												
SO_2_	kg	0.20	15.48				15.48	0.00				
H_2_SO_4_		0.07	0.00					63.20				63.20
NaOH (50%)		0.20	28.96				28.96	28.96				28.96
NH_3 _(25%)		0.27	2.36				2.36	1.68				1.68
H_3_PO_4 _(50%)		0.67	0.52				0.52	0.36				0.36
Defoamer		2.68	0.56				0.56	0.44				0.44
(NH_4_)2PO_4_		0.20	2.76				2.76	2.60				2.60
MgSO4.7 H2O		0.59	0.12				0.12	0.12				0.12
Enzymes	10^6 Filter paper unit	2.54	9.36				9.36	0.00				
Electricity-buy	MWh	40.13	0.18					0.18				
Cooling water	m^3^	0.02	72.48					65.44				
Process water	m^3^	0.19	3.36				3.36	3.20				3.20

**Employees**												
	Person	80,269										

**Co-products**												
Solid fuel	kg	0.11										
CO_2_		0										
Waste		0										

##### Adapting the reference processes to include pentose fermentation and straw as a feedstock

The 'reference processes' were adapted to include pentose fermentation using the following assumptions:

• The ethanol and carbon dioxide yield from pentose sugars was assumed to be 50%, reflecting the fact that the recovery of pentoses after pre-treatment is lower than for hexoses [26].

• The solid fuel yield and process heat requirement was unaffected.

• The flow of chemicals was the same as for the softwood enzymatic, or dilute acid, reference process.

• The capital cost of the plant was the same as for the softwood enzymatic, or dilute acid, reference process.

• Pentose fermenting yeasts were available at no additional cost; that is, although there would probably be some additional license costs, these would be negligible.

The 'reference' processes were adapted to use straw as a feedstock using similar assumptions:

• The ethanol and carbon dioxide yield from hexose sugars was assumed to be the same as for the softwood enzymatic, or dilute acid, reference process.

• The solid fuel yield and process heat requirement was unaffected.

• The flow of chemicals was the same as for the softwood enzymatic or dilute acid, reference process.

• The capital cost of the plant was the same as for softwood enzymatic or dilute acid reference process.

Modifying the reference process models in this way is clearly a significant simplification. Nevertheless, the approach permits an initial assessment of the relative importance of different process configurations within the context of the whole supply-chain. If comprehensive flow-sheet models were developed for each chain, a number of additional changes might be observed. These include: a reduction in the yield of solid fuel, or an increase in the demand for energy supplied externally to the process as the yield of ethanol increases. As part of the validation process, these assumptions, and the resulting mass balances, were discussed with the team that developed the original models and were moderated accordingly.

##### Incorporating scale effects

Returns-to-scale are anticipated. For the conversion plant returns-to-scale were estimated using the scale relationship: Cost B = Cost A (Scale B/Scale A)^*R *^[27]. For this approach, two pieces of information are necessary:

• the process components for which returns-to-scale can be expected; and,

• the appropriate scale factor (*R*) for each component.

Discussions with the team at Lund University suggested that the only area likely to experience returns-to-scale was the overall capital cost and that a reasonable estimate of the scale factor was *R *= 0.7, in line with literature estimates and engineering convention [27]. All other inputs to the process could be expected to vary in direct proportion to capacity (*R *= 1) with the exception of the number of employees, which would remain static (*R *= 0). For feedstocks, returns-to-scale are a function of the marginal cost of supply plus the additional transport cost of collecting material from a larger area. This iteration of the model incorporated a range of costs, but assumed that transport costs were constant.

##### Incorporating experience curves

The experience curve concept derives from the empirical observation that the unit costs of technology often decrease at a more or less fixed rate – the progress ratio (PR) – with every doubling of cumulative production. This idea was incorporated into mainstream economic literature by Arrow [28] in a review of 'the economic implications of learning by doing' and has been widely applied to the manufacturing sector [29]. Although for some technologies a progress ratio of 10% to 15% has been observed, the impact of accumulated experience on the cost of ethanol is expected to be less than this. This is because most elements of the conversion process, fermentation tanks, distillation columns, etc., and the feedstock supply, are already fully mature. When considering the impact of both experience curves and scale effects there is also a risk of double counting as one of the factors that may contribute to the learning rate is increasing returns-to-scale.

For these reasons, learning effects were only applied to two elements of the conversion process: the capital cost of pre-treatment and the unit price of enzymes. The PR estimated for the pre-treatment capital costs was 10%. This is comparable with the lower PRs observed for the Brazilian Proalcool programme: 7% between 1980 and 1985 and 29% between 1985 and 2002 [30]. This estimate might be considered pessimistic, but the price of pre-treatment reactors is, to a large extent, determined by the commodity price of corrosion-resistant stainless steel, which has been increasing. The PR estimated for the unit cost of enzymes was 30%. The justification for choosing a higher rate was that the current enzyme market is very small; consequently, significant scope for cost reduction may exist. Experience with established enzymes such as α-amylase also justifies this figure.

##### Cost of finance

The cost of financing a project reflects the perceived investment risk: consequently, the finance cost for a proven technology using an established plant design will be significantly less than for a first plant. The project finance variables included in the cost model are listed in Table 5.

**Table 5:** Project finance variables

Discount rate	The greater the discount rate, the more expensive it becomes to finance a project. For a project financed by a combination of debt and equity the effective discount rate is given by the Capital Asset Pricing Model. This discount rate is a function of the expected asset price volatility (Beta), risk-free market rate, market-risk premium, cost of debt and the ratio of debt to equity.
Investment life	A longer investment life increases the value of future revenues thereby reducing the cost of project finance.

Salvage value at end of project	The greater the salvage value, the lower the financing cost.

Capital grants	Capital grants directly reduce the amount of capital that must be financed by other means, thus lowering the finance cost.

Build duration	Increasing build time delays the point at which the project begins to generate revenues thereby increasing the financing cost.

Tax rate	Increasing the tax rate reduces the value of future revenues. This increases the cost of financing the project.

Depreciation	Proportion of capital costs which can be written off against tax each year; normally determined by legislation.

To assess the impact of variable financing costs, four project finance scenarios were developed: reference-case, first-plant, first-plant-with-capital-subsidy and *N*th-plant. The assumptions embedded within each scenario are listed in Table 6[31]. For all scenarios, insurance, maintenance and working capital were assumed to be constant fractions of fixed capital; investment life was also kept constant (15 years) and straight line depreciation over this lifetime was assumed. The reference-case was included because it is representative of the assumptions used for process comparison in previous techno-economic assessments [18,20]. This scenario assumed that the plant was built in 1 year and that the tax rate and salvage value were nil. For the other scenarios, the build cost was apportioned over 3 years and a constant tax rate and salvage value was assumed. The principal difference between the first-plant and *N*th-plant case is that the *N*th-plant has a lower cost of capital as a consequence of a greater proportion of debt finance and a lower value for Beta. The first-plant-with-capital-subsidy case is identical to the first-plant case with the addition of a 25% capital subsidy.

**Table 6:** Project finance scenarios

**Finance variable**	**Scenario**			
	Reference-case	First-plant	First-plant capital subsidy	*N*th plant – no subsidy
Beta		2.37^a^	2.37^a^	1.32^b^
Risk-free rate^c^		4.39%	4.39%	4.39%
Market-risk premium^d^		5%	5%	5%
Debt ratio = d/(e+d)		20%^e^	20%^e^	55%^f^
Cost of debt^g^		6%	6%	6%
Discount rate/cost of capital^h^	6%	14%	14%	7%
Investment life (years)	15	15	15	15
Salvage value at end of project (% initial investment)		5%	5%	5%
Investment grant (%)			25%	
Salvageable fraction of working capital at end of project (%)		5%	5%	5%
Build profile year: -2		20%	20%	20%
Build profile year: -1		50%	50%	50%
Build profile year: -0	100%	30%	30%	30%
Tax rate on net income		30%	30%	30%
Insurance – % fixed capital	1%	1%	1%	1%
Maintenance – % fixed capital	2%	2%	2%	2%
Working capital – % fixed capital	4%	4%	4%	4%

^a^Unlevered Beta, total for industry. Sector: petroleum producing. Source:

^b^Unlevered Beta. Sector: energy – alternate sources. Source:

^c^Determined via the interest rate of government bonds of a length equivalent to the investment useful life. Here we use the annual average yield from British Government Securities, 10 year nominal par yield of 4.4% (value at 31.12.2005, available on the Bank of England website section on 'statistics').

^d^UK average. Source:

^e^Estimated % debt obtainable for first-plant (personal communication fromUK Carbon Trust Venture Capital team).

^f^Estimated % debt obtainable for a grain-to-ethanol plant [31].

^g^Average cost of debt for petroleum producing sector 2005. Source Damodaran.com

^h^Calculated using the Capital Asset Pricing Model.

##### Estimating ethanol revenues and the cost of distribution, marketing and retail

To calculate the supply-chain NPV it is necessary to estimate net revenues from ethanol sales. Numerous factors may affect the price of ethanol: the price may be driven up by its contribution to fuel volume, octane enhancement value, the availability of tax credits, and its value as an oxygenate for regulatory compliance; the price may be driven down by the need for specialist handling requirements, impact on fuel volatility, energy content, impact on refinery production margins, etc. Historically, however, the basic pricing formula for ethanol producers in the US, where ethanol is sold as low (around 5–10%) percentage blends, attracts reduced duty levels compared with gasoline, and where the price at the pump is the same as regular gasoline, has been as follows [32]:

wholesale price = (gasoline price) + (value of subsidy) - (margin incentive)

Net revenues may then be calculated by subtracting the cost of distribution:

net ethanol revenue = (wholesale price) - (transport and distribution costs)

The consequence of this formula is that the price of a litre of ethanol more-or-less tracks the price of a litre of gasoline plus a premium deriving from policy incentives: that is, ethanol price = f(volume + subsidy). We assume that this pricing method may be applied to ethanol sold in Europe under current market conditions and use the label 'E5+subsidy' to refer to this pricing method in the discussion below.

If we conjecture that future ethanol supply was to increase significantly, two further scenarios are of interest.

• Ethanol continues to be sold as low percentage blends but the subsidy is removed; that is, the price of a litre of ethanol is equal to a litre of gasoline (price = f(volume)). This is assumed to correspond to ethanol sold as E5 (labelled 'E5').

• Ethanol is sold as high percentage blends and the price reflects its lower energy content; that is, the price is about two-thirds that of gasoline (price = f(energy content). This is assumed to correspond to ethanol sold as E85 with no subsidy (labelled 'E85').

Estimates used in the cost model are shown in Tables 7 and 8[33].

**Table 7:** Ethanol price determinants

**Variable**	**Value** (_2005_US$ litre^-1^)	**Comment**	**Reference**
Gasoline prices	0.43	Average EU wholesale price in 2005 (oil price of US$62 barrel^-1^.	[33]
	0.67	Estimated wholesale price for an oil price of US$100 barrel^-1^.	
	0.98	Estimated wholesale price for an oil price of US$150 barrel^-1^.	

Average value of subsidy^a^	0.28	Average difference between price of ethanol and gasoline. The principal assumption is that the price difference can be ascribed solely to EU subsidy regimes.	

Margin incentive^b^	0.027	Mid-point estimate for the US market.	[32]

Transport and distribution costs	0.032	Upper bound estimate for ethanol transport in US market.	[32]

^a^Ethanol: market fuel ethanol spot – FOB Rotterdam: 04.07.2006–07.08.2007. Gasoline price: unleaded – FOB Rotterdam barges: 04.07.2006–07/08/07.

^b^The margin incentive represents a discount to the purchaser to incentivise the use of ethanol compared with alternatives.

**Table 8:** Ethanol price scenarios

**Pricing method**	**Label**	**Net ethanol revenue **(_2005_US$ litre^-1^)		**Comment**
		**Oil price US$62 barrel^-1a^**	**Oil price US$100 barrel^-1^**	
			
Price = f(volume + subsidy)	E5+ subsidy	0.65	0.89	Price = (gasoline price) + (subsidy) - (margin incentive)) - (transport and distribution costs).

Price = f(volume)	E5	0.37	0.61	Price = (gasoline price) - (margin incentive)) - (transport and distribution costs).

Price = f(energy content)	E85	0.23	0.40	Price = (gasoline price) * (relative energy density) - (margin incentive)) - (transport and distribution costs).

^a^2005 EU average.

##### Normalising cost estimates

All costs have been converted to 2005 US$ using historic exchange rates from the year that the estimate was published, followed by conversion to 2005 values using the US all-commodities producer price index. US$ were chosen because oil is traditionally priced in dollars. In addition, the choice of dollars helps to minimise exchange rate errors and aids comparison with US studies; 2005 was chosen as the base year as this was the most recent period for which many complete datasets were available. It should be noted that oil prices have fluctuated dramatically over the past 3 years reaching a record $147 barrel^-1^in July 2008. At the time of writing the price of oil was around $100 barrel^-1^, considerably greater than its 2005 value ($62 barrel^-1^). The impact of oil price fluctuations is considered further in the sensitivity analysis.

##### Labelling individual supply-chains

Many hundreds of supply-chain permutations are possible. To clearly distinguish individual permutations the following labelling scheme is used:

Feedstock: softwood = spruce, straw = straw

Feedstock price: high = (H), medium = (M), low = (L)

Process: dilute acid = DA, enzymatic = EH, pentose fermentation = p

Finance: reference-case = (Rc), first-plant = (Fp), first-plant-with-subsidy = (FpS), *N*th plant = (Np)

Capacity: 25 odt.h^-1 ^= C(25)

For example, low-cost softwood processed using an enzymatic process including pentose fermentation, with a capacity of 25 odt h^-1^, financed as the *N*th plant, would have the label: 'spruce(L)-EHp(Np)-C(25)'. Where factors (for example, feedstock cost or scale) are identical, labels are included in the figure description.

## Results and discussion

This section presents a comparison of LE supply-chains developed from the components described above. The objective is to identify the elements of the supply-chain which have the greatest impact on the cost of ethanol production.

**Figure 3:**
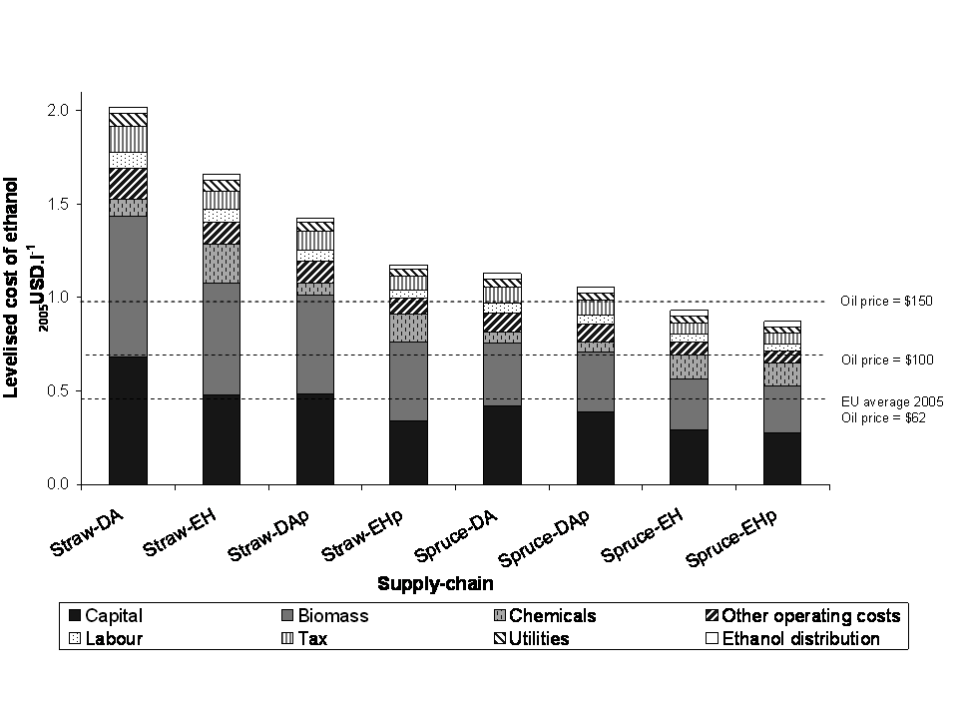
**Levelised cost of ethanol production**.

Base-case chains for comparison are defined in Table 9. The levelised cost of ethanol for each chain is shown in Figure 3. For all chains, the greatest contribution to the levelised cost is from biomass (31–40%) and capital (27–35%). It is also notable that as a proportion of total costs, the enzymatic process is characterised by a large cost of chemicals (15%), two-thirds of which can be attributed to the cost of enzymes alone.

**Table 9:** Base-case supply-chains for comparison

**Supply-chain label**	**Feedstock cost** (_2005_US$ odt^-1^)	**Finance**	**Ethanol pricing formula** (_2005_US$ odt^-1^)	**Capacity** (odt hour^-1^)
Straw-DA Straw-EH Straw-DAp Straw-EHp	Mid-range: 100			
		*N*th plant	Price = f(volume + subsidy)	25^a^
Spruce-DA Spruce-DAp Spruce-EH Spruce-EHp	Mid-range: 74			

^a ^A feed capacity of 25 odt hour^-1 ^corresponds to an ethanol output of approximately 35–55 million litres year^-1^.

Ethanol produced from straw is more expensive than ethanol from softwood for all base-case chains. This reflects the greater proportion of pentoses in straw compared with softwood, the lower efficiency of pentose fermentation, and the lower cost estimate for softwood feedstocks. A notable consequence of this difference is that the introduction of pentose fermentation reduces the cost of production from straw by around 29%, whereas it only reduces the cost of production from softwood by around 8%. At this scale, the lowest cost supply-chain (spruce-EHp) produces ethanol roughly twice as expensive as the 2005 average cost of gasoline ($0.43 litre^-1^). The most expensive supply chain (straw-DA) produces ethanol nearly four and a half times more expensive.

### The impact of increasing plant capacity

**Figure 4:**
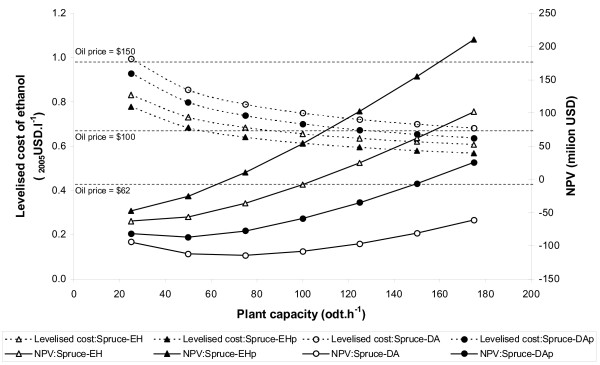
**Variation in supply-chain cost performance with plant capacity**. Softwood base-case supply chains. Net present value calculated using an oil price of US$62 barrel^-1^.

**Figure 5:**
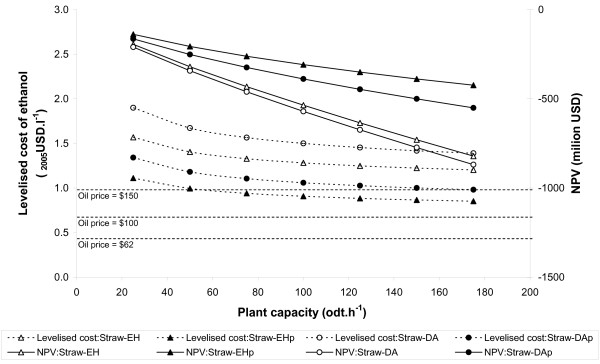
**Variation in supply-chain cost performance with plant capacity**. Straw base-case supply-chains. Net present value calculated using an oil price of US$62 barrel^-1^.

Figures 4 and 5 show the variation in levelised cost and NPV as plant size increases. For softwood, all the chains demonstrate positive returns to scale: the larger the plant the more profitable it is and the lower the production cost. The levelised cost converges towards around $0.6–0.7 litre^-1^as capacity increases and, for the enzymatic process, it equals the $100 barrel^-1 ^gasoline price at a plant capacity of around 60–100 odt hour^-1 ^(around 60–120 million litres year^-1 ^ethanol). It is notable that changes in scale have a proportionately greater impact than switching between enzymatic and dilute acid hydrolysis, or including (excluding) pentose fermentation.

For straw, the difference between chains is greater than for softwood. Again, this reflects the greater proportion of pentoses in straw compared with softwood and the lower efficiency of pentose recovery and fermentation. Although the levelised cost decreases with increasing capacity, it converges towards around $1.0–1.5 litre^-1^, nearly twice the $100 barrel^-1^gasoline price. With the 2005 average oil price ($62 barrel^-1^), the NPV range is negative for all straw chains and scales.

### The impact of varying ethanol revenues

**Figure 6:**
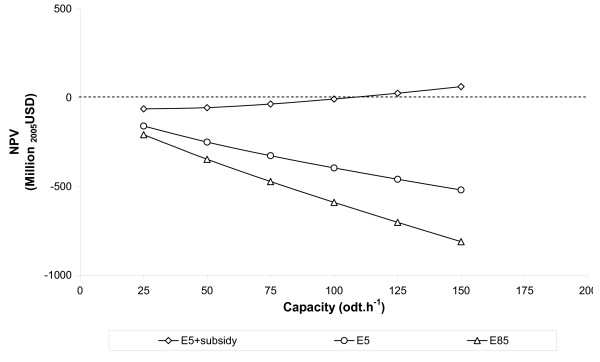
**Variation in supply-chain net present value with ethanol revenue and capacity for an enzymatic hydrolysis plant**. The example shown excludes pentose fermentation and assumes a mid price feedstock (US$74 odt^-1^), *N*th plant finance and an oil price of US$62 barrel^-1^.

Supply-chain NPV is very sensitive to the net revenues obtained from ethanol sold: the greater the revenues the greater the NPV. Figure 6 shows the impact of varying the ethanol costing method on the supply-chain NPV of a softwood enzymatic-hydrolysis plant.

For this chain, only the case in which ethanol is priced as a function of volume plus subsidy (E5 + subsidy) is profitable and this would require a plant capacity in excess of 100 odt hour^-1 ^(around 220 million litres ethanol year^-1^).

Where ethanol is priced as a function of volume (E5) or energy content (E85) (see Table 8), returns-to-scale arising from increased capacity are not sufficient to make the supply-chain profitable. Although not shown, similar relationships may be observed for other supply-chain configurations.

The methodology for pricing ethanol will ultimately depend upon the size of the total fuel ethanol market, the political priority given to the support of alternative fuels and the availability of politically controlled incentives such as carbon credits. An assessment of political risk will therefore be highly influential on an investor's decision to build a lignocellulosic ethanol plant.

### The impact of finance scenarios and experience curves

**Figure 7:**
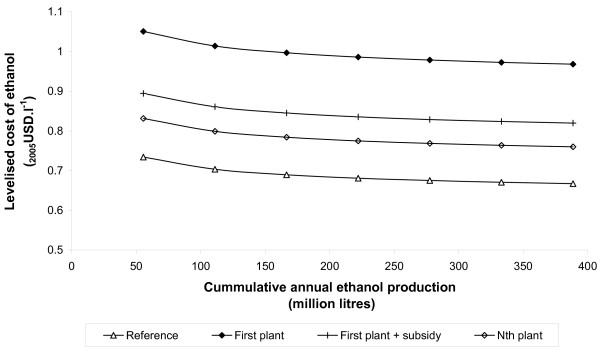
**Variation in levelised cost with cumulative annual production and finance scenarios**. The example shown is for a softwood enzymatic hydrolysis plant, excluding pentose fermentation, and assuming a mid price feedstock (US$74 odt^-1^).

Figure 7 shows the variation in the levelised cost of ethanol with increasing cumulative capacity. The successive introduction of seven plants is shown, each with a capacity of 25 odt hour^-1 ^and assuming the PR and finance scenarios described above. Again, the results shown are for the softwood enzymatic chain, but similar relationships were observed for the other supply-chain configurations. In this case, cost reductions attributable to learning effects are around 2% to 3%, but the change in cost attributable to moving from first-plant to *N*th plant finance scenarios is around 20%, nearly an order of magnitude greater. The progress ratios considered here may, perhaps, be considered conservative; nevertheless, it is apparent that the benefit of obtaining favourable finance terms has the potential to dwarf the benefit obtained from small reductions in capital cost.

### Sensitivity analysis

The relationships shown above clearly show that ethanol cost estimates are influenced by a great number of assumptions. Testing the stability of the model output against variation in these assumptions is important to identify which have the greatest influence. In this analysis, the testing was broken down into two steps.

In the first step, an elasticity analysis was conducted on each of the parameters feeding into the model. The elasticity of a result with respect to an input parameter is defined as the ratio of the percentage change of the result to the percentage change in the parameter. A small change, much less than 1, denotes an inelastic parameter – one that is forgiving of small uncertainties. Whereas an elasticity close to 1 shows that the parameter has a greater influence on the model result and indicates that a more accurate input is required.

In the second step, the parameters identified as important were varied over a range of values and the change in results recorded. The outcome is presented graphically in the form of a spider diagram showing the change in the result as a function of the percentage change in the parameters.

The elasticity of the supply-chain NPV performance metric was calculated with respect to all cost parameters and for all the supply chains. For clarity, only parameters with elasticity greater than 0.01 are shown, and are listed in Table 10.

**Table 10:** Cost parameters included in the sensitivity analysis

**Parameter**	**% Variation relative to base-case**		**Remark**
		
	**Minimum**	**Maximum**	
Fixed capital investment	0.7	1.3	A range of +/- 30% was considered sufficient to cover uncertainties in capital cost.

Cost of biomass	0.6	1.7	A range of -40% to +70% approximates to the 15th and 85th percentiles obtained in the survey of EU cost estimates for softwood and straw.

Enzymes	0.5	1.5	A range of +/- 50% was considered reasonable given the uncertainties in the costs of enzyme production.

Cost of ethanol	0.3	1.85	The minimum -70% reflects the price of ethanol valued on an energy basis with an oil price of US$40 barrel^-1^. The maximum +85% reflects ethanol valued on a volume basis plus subsidy, assuming an oil price of US$150 barrel^-1^.

Solid fuel revenue	0.7	1.3	A range of +/- 30% was considered sufficient to cover uncertainties in the retail price of solid fuel.

Distribution cost	0.7	1.3	A range of +/- 30% was considered sufficient to cover uncertainties in distribution cost.

Effective discount rate	1	2	A discount rate range of 7–30% was considered sufficient to cover uncertainties in how a plant may be financed.

**Figure 8:**
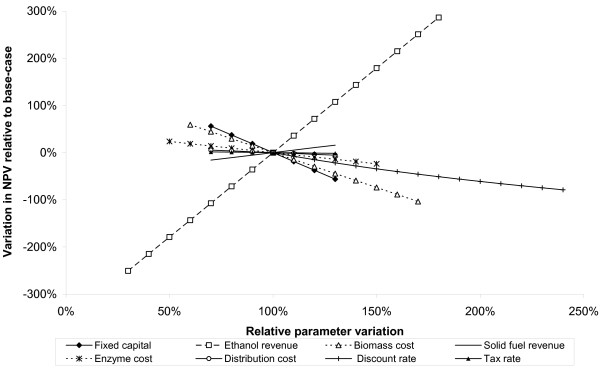
**The sensitivity of supply-chain net present value to changing input parameter assumptions**. The example shown is for the softwood enzymatic process spruce(M)-EH(Np)-C(25).

The results of the sensitivity analysis are similar for all chains. An illustrative spider diagram showing the results for the softwood enzymatic process assuming a 25 odt hour^-1 ^(around 55 million litres year^-1^) plant is shown in Figure 8. Lines that rise from the left to the right show a positive correlation between the result and a change in input values and vice versa. The gradient indicates elasticity: the steeper the line, the greater the impact that changing that parameter will have on the result. The length of the line indicates the range of values that the parameter may take. For the example shown, the parameters with the greatest influence on supply chain NPV are ethanol revenues, feedstock price and discount rate. These parameters may take a wide range of possible values and have elasticities close to 1. In contrast, the cost of enzymes is highly uncertain, but because the elasticity is lower, varying this parameter has less impact on the overall result. The capital cost of the plant is negatively correlated with NPV and has a high elasticity (around 0.7). This parameter varies over a limited range of values, however, suggesting that incremental reductions in capital cost are less important than securing a low cost supply of feedstocks and obtaining a high value for ethanol. Although not shown, if the capacity of the plant is increased, the main effect is to reduce the sensitivity to changes in capital cost.

**Figure 9:**
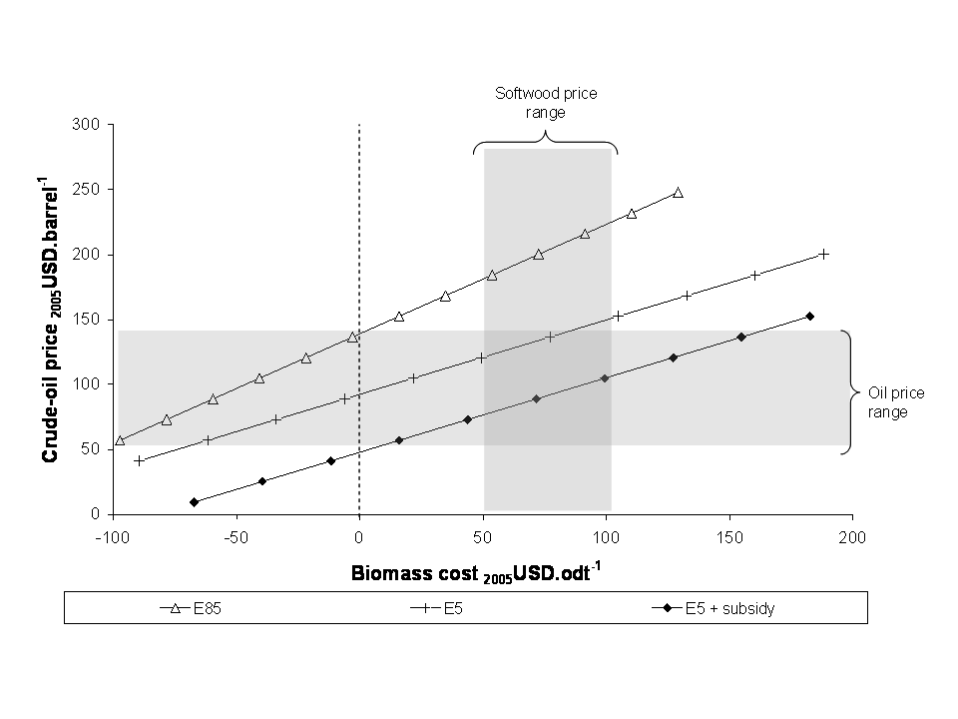
**Break-even oil and feedstock prices for base-case softwood chains**. Spruce-EH(Np)-C(25).

**Figure 10:**
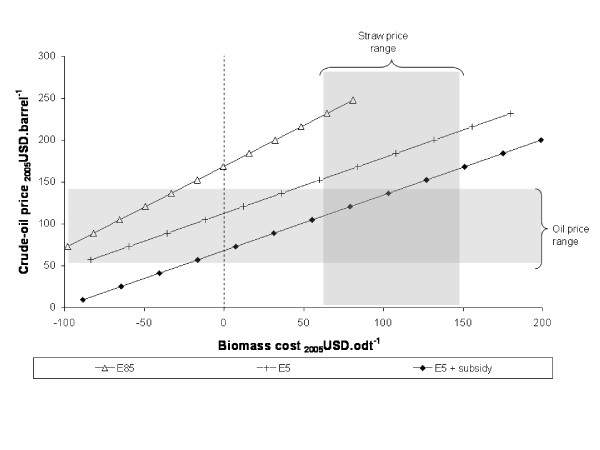
**Break-even oil and feedstock prices for straw supply-chains incorporating pentose fermentation**. Straw-EHp(Np)-C(25).

In identifying which assumptions have the greatest influence on model outputs, the sensitivity analysis results help prioritise further research: those parameters with the greatest influence merit the greatest attention. From the results shown, it is evident that the relationship between the cost of feedstocks and the value obtained for ethanol is particularly important to determining commercial viability. This relationship is shown explicitly in Figures 9 and 10 for the base-case softwood chain and for the straw chain including pentose fermentation. On each figure, the three lines correspond to the three ethanol pricing methods; a single point on one of the lines corresponds to the oil and biomass prices at which that particular supply-chain would break-even (NPV = 0); typical oil and biomass price ranges are indicated by the shaded areas.

For the softwood example, it can be seen that the E5 and E5+subsidy chains break-even at moderate oil and biomass prices, but that the E85 chain would require either very low feedstock prices or very high oil prices. For the straw case, the E5+subsidy chain breaks even at high oil (low biomass) prices but the E5 and E85 chains fall outside the typical price ranges shown. Although not shown, similar relationships can be demonstrated for the other supply chains. Factors which decrease the cost of production – for example, more favourable finance, increased capacity, or improved yield of ethanol relative to solid fuel – shift the lines downwards. Factors which increase the cost of production have the opposite effect.

## Conclusion

The model described here has been used to characterise and compare simplified ethanol supply-chains applicable to Europe. Using this model it has been possible to identify which factors are most important in determining the cost and profitability of ethanol and compare a range of supply-chain configurations. To a limited extent it was also possible to assess the relative impact of process variations and improvements such as pentose fermentation on supply-chain cost performance.

The results suggest that ethanol produced from softwood and sold as a low percentage blend with gasoline could be cost competitive with gasoline without requiring subsidy. Production from straw, however, would generally be less competitive, owing to the greater proportion of less easily fermentable pentose sugars. For the same reason, the benefit of introducing pentose fermentation would be greatest for the straw processes. The commercial attractiveness, however, is by no means certain. The most important factors affecting commercial viability are the cost of feedstocks, primarily determined by location and existing markets, and the value obtained for ethanol, primarily determined by oil price and policy incentives. Both of these factors are highly uncertain.

Feedstock markets are diverse and generally geographically constrained, reflecting the low density of most biomass materials. Consequently, it would be misleading to present straw and softwood as competing options from a feedstock or process development perspective. They are produced in different regions and are subject to different market pressures.

The value obtained for ethanol is determined not only by the oil price and policy framework, but also by the percentage blend at point of sale. Ethanol has a lower value when sold on the basis of its energy content, in high percentage blends, compared with its sale on the basis of its oxygenate value in low percentage blends. Taking the market as a whole, therefore, there appears to be no commercial logic in seeking to sell ethanol as high percentage blends whilst the market for low percentage blends remains unsaturated. Similarly, the cost of policy interventions seeking to promote the use of ethanol through the uptake of high percentage blends would be proportionately more expensive than those seeking to extend the use of low percentage blends.

Looking at the market from the perspective of an individual ethanol producer, high percentage blends may nevertheless be attractive. Particularly, if they allow the ethanol producer to capture a greater proportion of the value chain, or if regional policies prioritise local environmental benefits.

Capital costs are also important, and because of increasing returns to scale, the larger the processing plant the more profitable it will be. In this regard, the economics of lignocellulosic ethanol production might be considered analogous to the economics of pulp production, the pulp industry having demonstrated a trend towards larger plant and more centralised production in areas where feedstocks are abundant. From a technology development perspective, an alternative development pathway that does not require a large stand-alone plant and the associated initial investment would obviously be advantageous. Although not considered here, this might be achievable if ethanol production was integrated with other facilities such as combined heat and power, fossil fuel refineries (for access to utilities) or existing pulp mills; or in an arable region, from integrating production from grain and straw. A subject for future study could be to investigate the dependence of facility capital cost on geographic location (rather than simple production capacity).

On the basis of the results presented, we may conclude that supply-chain design will play a critical role in determining whether lignocellulosic ethanol production is commercially viable. The importance of feedstock supply highlights the need for location-specific assessments of feedstock availability and price. Similarly, the role of subsidies and policy incentives in creating and sustaining the ethanol market highlights the importance of political engagement, and the need to include political risks in investment appraisal.

Further technical improvements to drive down costs and improve the yield of the highest value products are, of course, still needed. The modelling tool developed here is complementary to existing flow-sheeting and experimental methods and, by enabling the rapid comparison of supply chains at the systems level, extends the scope of assessment. As the technology gets closer to market and the need for large-scale public and private investment increases, it is to be hoped that broadening the scope of assessment in this way will serve to improve the understanding of how support can be delivered and deployment achieved.

## Abbreviations

C: capacity; DA: dilute acid conversion process; EH: enzymatic conversion process; Ehp: enzymatic conversion process incorporating pentose fermentation; Fp: first-plant; FpS: first-plant with subsidy; LE: lignocellulosic ethanol; NILE: New Improvements in Lignocellulosic Ethanol (an EU Framework Programme 7 project); Np: *N*th plant; NPV: net present value; NREL: National Renewable Energy Laboratory; odt: oven-dry-tonne; PR: progress ratio; SHF: separate hydrolysis and fermentation; SSF: simultaneous saccharification and fermentation

## Competing interests

The authors declare that they have no competing interests.

## Authors' contributions

RS designed and executed the study including model development, results analysis and drafting of the manuscript. AB conceived the study and both AB and NS participated in the study design. All authors read, commented upon and approved the final manuscript.
